# miR-550a-5p Functions as a Tumor Promoter by Targeting LIMD1 in Lung Adenocarcinoma

**DOI:** 10.3389/fonc.2020.570733

**Published:** 2020-10-28

**Authors:** Zi-Zhang Guo, Zi-Jian Ma, Yao-Zhou He, Wei Jiang, Yang Xia, Chun-Feng Pan, Ke Wei, Yi-Jun Shi, Liang Chen, Yi-Jiang Chen

**Affiliations:** Department of Thoracic and Cardiovascular Surgery, The First Affiliated Hospital of Nanjing Medical University, Nanjing, China

**Keywords:** lung adenocarcinoma, microRNA, oncogene, cancer therapy, cell proliferation

## Abstract

Lung adenocarcinoma accounts for half of all lung cancer cases in most countries. Mounting evidence has demonstrated that microRNAs play important roles in cancer progression, and some of them can be identified as potential biomarkers. This study aimed to explore the role of miR-550a-5p, a lung adenocarcinoma-associated mature microRNA screened out from the TCGA database via R-studio and Perl, with abundant expression in samples and with 5-year survival prognosis difference, as well as having not been studied in lung cancer yet. Potential target genes were predicted by the online database. Gene ontology enrichment, pathway enrichment, protein–protein interaction network, and hub genes–microRNA network were constructed by FunRich, STRING database, and Cytoscape. Then, LIMD1, a known tumor suppressor gene reported by multiple articles, was found to have a negative correlation with miR-550a-5p. The expression of miR-550a-5p was up-regulated in tumor samples and tumor-associated cell lines. Its high expression was also correlated with tumor size. Cell line A549 treated with miR-550a-5p overexpression promoted tumor proliferation, while H1299 treated with miR-550a-5p knockdown showed the opposite result. Mechanically, miR-550a-5p negatively regulated LIMD1 by directly binding to its 3′-UTR validated by dual luciferase assay. In summary, a new potential prognostic and therapeutic biomarker, miR-550a-5p, has been identified by bioinformatics analysis and experimental validation *in vitro* and *in vivo*, which promotes lung adenocarcinoma by silencing a known suppressor oncogene LIMD1.

## Introduction

Lung cancer is the most dangerous disease and is thought of as the foremost cause of cancer-related death around the world. The 5-year survival prognosis of lung cancer patients is generally <15% (1). Among them, lung adenocarcinoma (LUAD) accounts for half of all lung cancer cases in most countries (2). Therefore, specific molecules serve as biomarkers for early diagnosis, and therapeutic targets in lung cancer become urgently needed.

microRNAs (miRNAs) are very short non-coding RNAs (20–24 nucleotides) that were discovered in 1993 (3) and expeditiously recognized that miRNAs are involved in multiple aspects of lung cancer such as cell proliferation, apoptosis, invasion, and EMT (4). Now, it was well recognized that apart from few studies that have unconcealed the role of miRNAs in activating gene expression in specific conditions, most miRNAs can identify and bind to complementary sites present in the 3′-untranslated region (UTR) of target mRNA resulting in post-transcriptional gene silencing (5).

The rise of bioinformatics analysis technology enables people to analyze genetic data in various databases. Based on the Cancer Genome Atlas (TCGA) database, we screened out LUAD-associated miRNAs by R-studio and Perl, with different expression and with 5-year survival prognosis, as well as having not been reported in lung cancer-associated articles. Other bioinformatics analysis technologies like GO enrichment, pathway enrichment, protein–protein interaction (PPI) network, hub gene–miRNA, and so on were performed for further exploration.

The present study aims to explore and identify LUAD-associated differential expression miRNAs (DE-miRNAs) and their potential molecular mechanisms through a series of bioinformatics analyses. Then, experiments *in vitro* and *in vivo* were carried out to verify the consistency with hypothesis based on informatics analysis.

## Materials and Methods

### Patients and Tissue Collection

Twenty-nine pairs of NSCLC and matching adjacent normal tissues were obtained from NSCLC patients who underwent thoracoscopic surgery at the First Affiliated Hospital of Nanjing Medical University from January 2019 to December 2019. All specimens were preserved in liquid nitrogen after resection until use. Informed consent was acquired from patients. This current research was approved by the Department of Ethics Committee of our hospital.

### Bioinformatics Analysis

TCGA database (https://portal.gdc.cancer.gov/) was used to download vital biomarker miRNA in LUAD. Perl (version 5.28) and R-studio (version 3.6) were used to selected suitable differential miRNAs (*P*_adj_ = 0.01, fold change = 2). Then, heatmap, volcano, and survival analysis were drawn. Target genes of relative miRNAs were predicted by miRtarBase (http://mirtarbase.mbc.nctu.edu.tw). GO enrichment analysis, pathway enrichment analysis, PPI network analysis, and hub genes analysis were performed by Enrichr (http://amp.pharm.mssm.edu/Enrichr), STRING database (https://string-db.org), FunRich (version 3.13), and Cytoscape (version 3.8). An online survival analysis tool Kaplan–Meier Plotter database (https://kmplot.com/analysis) was used to further evaluate target genes.

### Cell Culture

Five human LUAD cell lines (SPCA1, A549, H358, PC9, and H1299) and one human bronchial epithelioid cell line (16HBE) were involved in this study. All cell lines above were bought from the American Type Culture Collection. Cells were cultured in RPMI-1640 medium + 10% fetal bovine serum along with penicillin (100 U/ml) and streptomycin (100 μg/ml, Invitrogen, Carlsbad, CA) in an incubator containing 5% CO_2_ at 37°C.

### Cell Transfection

Lentiviral (Lv-miR-550a-5p, Sh-miR-550a-5p, Lv-vector, Sh-vector), plasmid-LUAD, and siRNA-LIMD1 were purchased from Gene Pharma (Shanghai, China). Selected cell lines were infected with lentiviral vectors and screened with puromycin according to the protocols. Lipofectamine 3000 (Invitrogen) was used for plasmid-LIMD1 and siRNA-LIMD1 transfection.

### RNA Extraction and Real-Time Quantitative PCR (RT-qPCR)

TRIzol reagent (Invitrogen) was used to isolate total RNA from tissues and cells based on the instructions. cDNA was generated from total RNA using the PrimeScript RT reagent (Takara, Japan), and RT-qPCR was performed with SYBR Green Master Mix II (Takara) on an ABI 7900 fast real-time PCR system (ABI, CA, USA). Data of mRNA and miRNA were normalized by GAPDH and U6. All the experiments were performed three times independently. The oligonucleotides used in this study are shown in Table 1.

**Table 1:** Sequences of the primers in this study.

**Name**	**Sequence (5′ → 3′)**
miR-550a-5p F	TGCTGTTAGGTTGTCTTCA
miR-550a-5p R	CTATGTTTTGTCCAATTTCT
U6 F	CTCGCTTCGGCAGCACATATACT
U6 R	ACGCTTCACGAATTTGCGTGTC
GAPDH F	AAGGTGAAGGTCGGAGTCA
GAPDH R	GGAAGATGGTGATGGGATTT
LIMD1 F	TGGGGAACCTCTACCATGAC
LIMD1 R	CACAAAACACTTTGCCGTTG
miR-550a-5p inhibitor	GGGCUCUUACUCCCUCAGGCACU
miR-550a-5p inhibitor NC	CAGUACUUUUGUGUAGUACAA
miR-550a-5p mimics	AGUGCCUGAGGGAGUAAGAGCCC
miR-550a-5p mimics NC	UUCUCCGAACGUGUCACGUTT
si-LIMD1	GGGCCCAAAUCUUACCUUUTT
si-NC	UUCUCCGAACGUGUCACGUTT

*F, forward primer; R, reverse primer*.

### CCK-8 Assay

Transfected NSCLC cells (1 × 10^3^ cells/well) were incubated into the plates (96-well). Three replicate wells were used for each group. CCK-8 solution (Beyotime, Shanghai, China) was added into each well (10 μl/well) for 2 h of incubation. Finally, the absorbance was examined at 450 nm (A450) by using a spectrophotometer (Thermo Scientific, Rockford, IL, USA).

### The 5-ethynyl-2′-deoxyuridine (EdU) Assay

Regent EdU Apollo567 *in vitro* Flow Cytometry Kit (Ribobio, Guangzhou, China) was used to determine cell proliferation capacity. Cells were incubated with EdU (50 μm) for 2 h. Positive cells were screened by Apollo and DAPI staining through fluorescence microscope. The ratio of EdU-positive to total DAPI-positive cells representing the EdU incorporation rate.

### Colony Formation Assay

Results were displayed via the clonogenicity of a single cell. In brief, the transfected cells were seeded into 60-mm plates (1 × 10^3^ cells/plate). The culture medium was changed every 5 days. After 10 days, cells were washed with PBS twice and then fixed and stained with crystal violet staining solution (Beyotime, Shanghai, China) for 15 min. Colonies containing ≥50 cells were counted. Each experiment was performed independently three times.

### Flow Cytometric Analysis

In the cell cycle experiment, the cells were harvested and 70% pre-cooled ethanol was added for times ranging from 2 h to overnight, and then the cells were stained with propidium iodide (PI) (Vazyme, Nanjing, China) by FACScan flow cytometry for 30 min, while cell apoptosis was evaluated with an APC/7-AAD apoptosis detection kit (KeyGen Biotech Co., Ltd., Nanjing, China) according to the manufacturer's instructions.

### Western Blotting

Total protein of cells was extracted from RIPA buffer (Beyotime, Shanghai, China) containing 100 μg/ml PMSF (Beyotime, Shanghai, China) and 2 μg/ml aprotinin (Beyotime, Shanghai, China). Protein lysates were separated by 10% SDS-PAGE and transferred to the PVDF membrane. After that, the membrane was blocked by 5% BSA for 2 h and then incubated with primary antibodies and secondary antibody. Primary antibodies used in Western blotting were LIMD1 (Cell Signaling Technology, 13245), Ki67 (Abcam, ab92742), and GAPDH (Cell Signaling Technology, 5174).

### Dual-Luciferase Assay

The 3′-UTR sequence or the mutant sequence of LIMD1 was cloned into pGL3 promoter vector (Genscript, Nanjing, China). A549 cells were cultured in 24-well plates transfected miR-550a-5p mimics or negative control (NC). Then, cells were transfected with pGL3-LIMD1 3′-UTR or pGL3-LIMD1-MUT by Lipofectamine 3000 reagent (Invitrogen). Twenty-four hours later, the cells were collected and Renilla luciferase activity was considered as a normalization using Dual-Luciferase Assay Kit (Promega).

### The Xenograft Model

With the approval of the Animal Care and Use Committee of Nanjing Medical University, *in vivo* experiments were performed with a group of BALB/C nude mice (4–5 weeks old) purchased from the Animal Center of Nanjing Medical University. Subsequently, mice were randomly divided into four groups (five mice/group). Both flanks of mice were subcutaneously injected with H1299 cells or A549 cells stably down-regulating or up-regulating miR-550a-5p or NC. Tumor size (calculated as length × width × 0.5 mm^3^) was measured every 5 days. After 4 weeks, nude mice were euthanized and nodules were weighed.

### Immunohistochemistry (IHC) Analysis

All samples were fixed with 4% formalin solution and embedded in paraffin. Then, 5-μm-thick sections were made and incubated with Ki-67 primary antibody (Abcam, 92742) overnight at 4°C. The next day, after being washed with PBS, sections were incubated with an HRP-conjugated secondary antibody for 1 h at 37°C. Then, DAB solution was used for staining for 5 min, accompanied by hematoxylin for counterstaining. Images were evaluated by an Olympus microscope (Olympus, Tokyo, Japan).

### Statistical Analysis

All experiment data were analyzed through GraphPad Prism (version 8.0) and SPSS (version 19.0). *P*-values were analyzed using Student's *t*-test, and *P* < 0.05 was regarded as significant.

## Results

### Identification of DE-miRNAs

**Figure 1:**
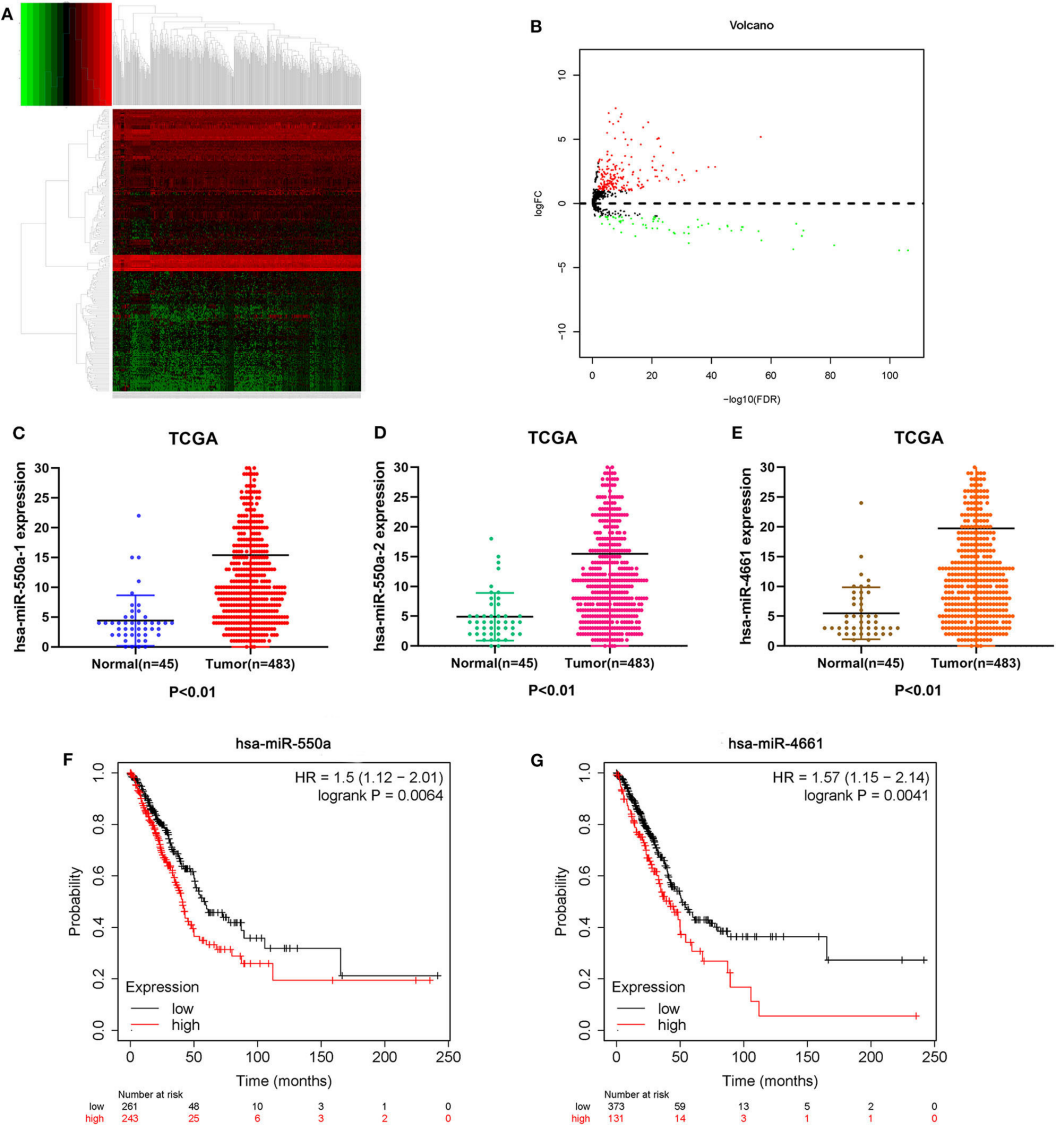
Identification of DE-miRNAs. **(A)** Heatmap of DE-miRNAs. The black patterns represent miRNAs that are not differentially expressed between 483 human LUAD tumor samples and 45 human normal samples. The red patterns represent up-regulated miRNAs and the green represent down-regulated miRNAs. **(B)** Volcano plot of DE-miRNAs. The black plots represent miRNAs with no difference, while the red plots represent up-regulated miRNAs and the green plots represent down-regulated miRNAs. **(C–E)** The expression of miR-550a-1, miR-550a-2, and miR-4661 in tumor tissues was compared with that in normal tissues in LUAD patients from TCGA database. **(F,G)** Survival curve of hsa-miR-550a and hsa-miR-4661 in LUAD from the Kaplan–Meier database.

Data from 528 patient samples, consisting of 483 tumor samples and 45 normal samples, were downloaded from the TCGA database. “Bronchus and lung,” “TCGA-LUAD,” “adenomas and adenocarcinomas,” “miRNA-Seq,” “primary tumor,” “solid tissue normal,” “transcriptome profiling,” and “miRNA Expression Quantification” were keywords selected to search in the repository. Subsequently, differential expression analysis was conducted via R-studio and Perl, and DE-miRNAs including 212 up-regulated and 67 down-regulated miRNAs were obtained (|logFC1| ≧ 1, *P* value <0.01). A volcano plot and a heatmap of these DE-miRNAs are displayed in Figure 1. To screen out miRNAs with research value, the DE-miRNAs reported in LUAD-related articles and with no significant difference in 5-year survival prognosis were ignored. Therefore, miR-550a-1, miR-550a-2, and miR-4661 were taken into consideration (Figure 1; Table 2).

**Table 2:** The DE-miRNAs screened from TCGA database.

**miRNA**	**Log_**2**_FC**	**Log_**2**_CPM**	***P* value**	**FDR**
hsa-mir-550a-1	1.26904869	1.763440688	2.46E−11	1.27E−10
hsa-mir-550a-2	1.111075555	1.794780258	1.59E−08	6.06E−08
hsa-mir-4661	1.292346831	2.174747025	1.18E−08	4.54E−08

### Go Functional and Biological Pathway Enrichment Analysis

It is well known that pre-miRNA will evolve into two mature single strands through the enzyme digestion reaction. The passenger strand is often degraded, while the guide strand combines with Ago2 protein to form RNA-induced silencing complex (RISC). Mature guiding strands miR-550a-5p derived from miR-550a-1 or miR-550a-2 and miR-4661-3p derived from miR-4661 were selected. Then, 134 potential target genes were predicted for the two selected up-regulated mature miRNAs by using miRTarBase. Subsequently, GO functional annotation analysis was conducted, including molecular function (MF), biological process (BP), and cellular component (CC).

**Figure 2:**
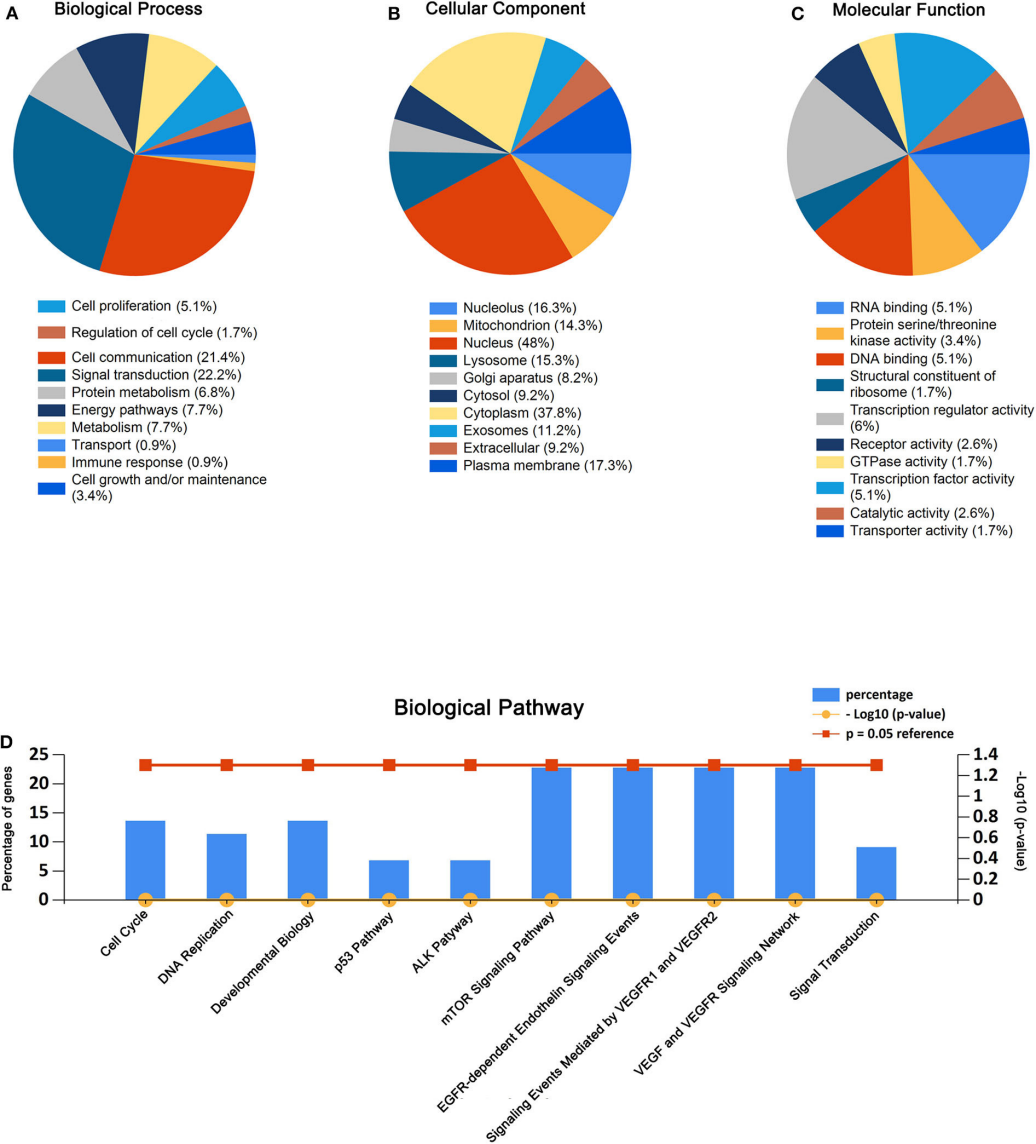
GO enrichment analysis and pathway enrichment analysis for the predicted target genes of miR-550a-5p and miR-4661-3p. **(A–C)** Enriched biological process, cellular component, and molecular function of the target genes. **(D)** Pathway enrichment analysis of the target genes. The blue bars represent percentage of the target genes, and the red line and the yellow line represent *P*-value (0.05) and –log_10_ (*P*-value), respectively.

As presented in Figure 2, the enriched GO functions for the potential target genes included cell growth, signal transduction, cell communication, cell proliferation, regulation of nucleobase in the BP; plasma membrane, extracellular, exosomes, cytoplasm exosomes in the CC, transporter activity, catalytic activity, RNA binding, transcription regulator activity, and receptor activity in the MF. These results were analyzed by FunRich.

Similarly, biological pathway enrichment analysis of the target genes was also performed by FunRich. The enriched pathways contained signaling events mediated by VEGFR1 and VEGFR2, VEGF and VEGFR signaling network, ALK1 pathway, cell cycle, mitotic, p53 pathway, and mTOR signaling pathway (Figure 2).

All these results hinted that miR-550a-5p and miR-4461-3p might affect LUAD via modulating cell proliferation.

### PPI Network and miRNA-hub Gene Network

**Figure 3:**
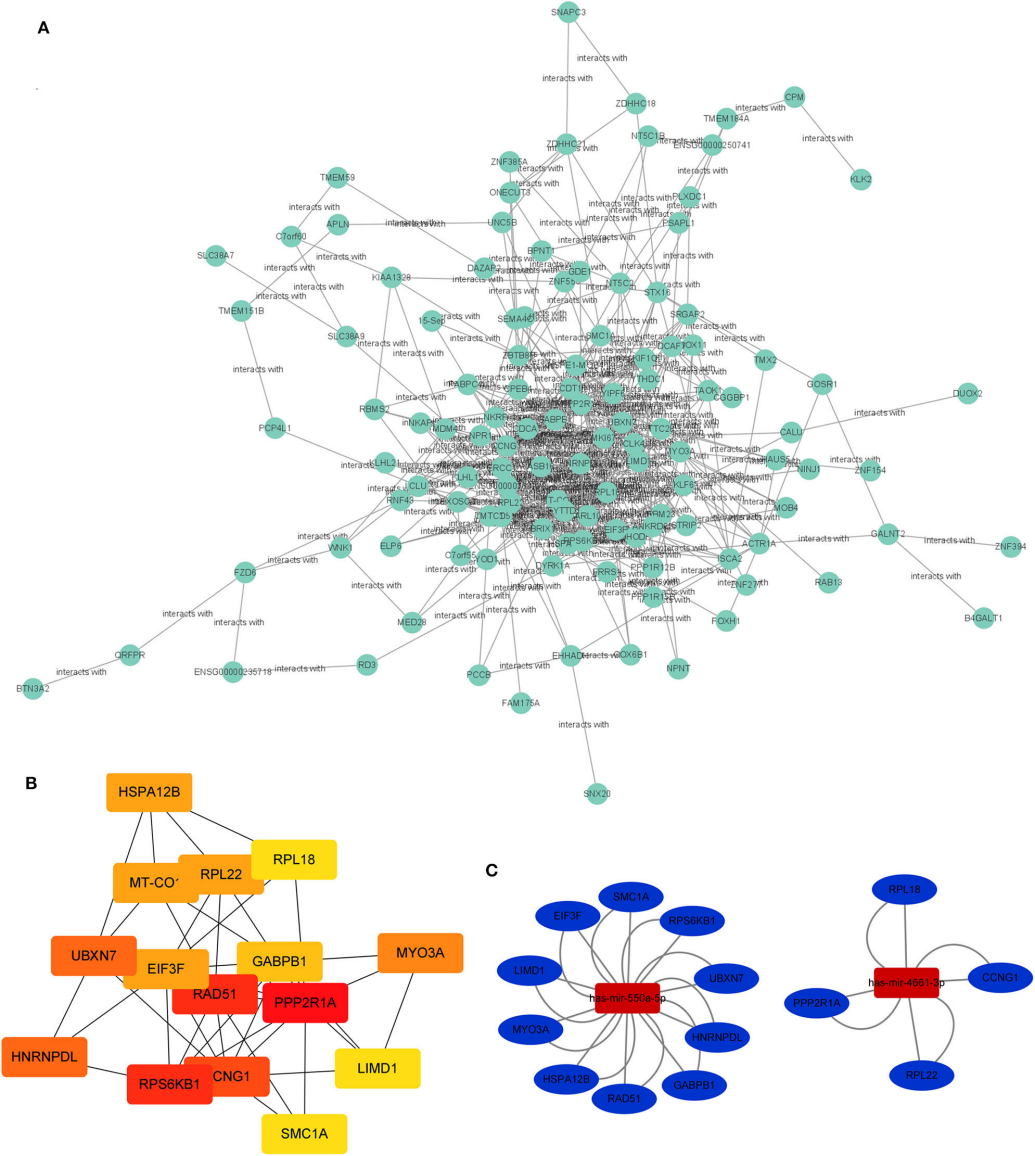
PPI network and miRNA-hub gene network. **(A)** Protein–protein interaction network of the target genes. **(B)** Hub genes identified in the PPI interaction. The darker the color, the more important the gene. **(C)** The regulatory network between the selected miRNAs and hub genes.

The PPI network of target genes of miR-550a-5p and miR-4461-3p was drawn by Cytoscape, revealing interaction among these target genes (Figure 3). The matching nodes of target genes were constructed via the STRING database. Since, in the PPI network, the more genes a certain gene can affect, the more important this gene is, 15 hub genes were analyzed by cytohubba function in Cytoscape according to node degree.

Subsequently, the miRNA-hub gene network was constructed (Figure 3). Ten hub genes could be modulated by miR-550a-5p, and five hub genes could be regulated by miR-4661-3p. This network suggested that miR-550a-5p and miR-4661-3p might be the key potential regulator in LUAD.

**Figure 4:**
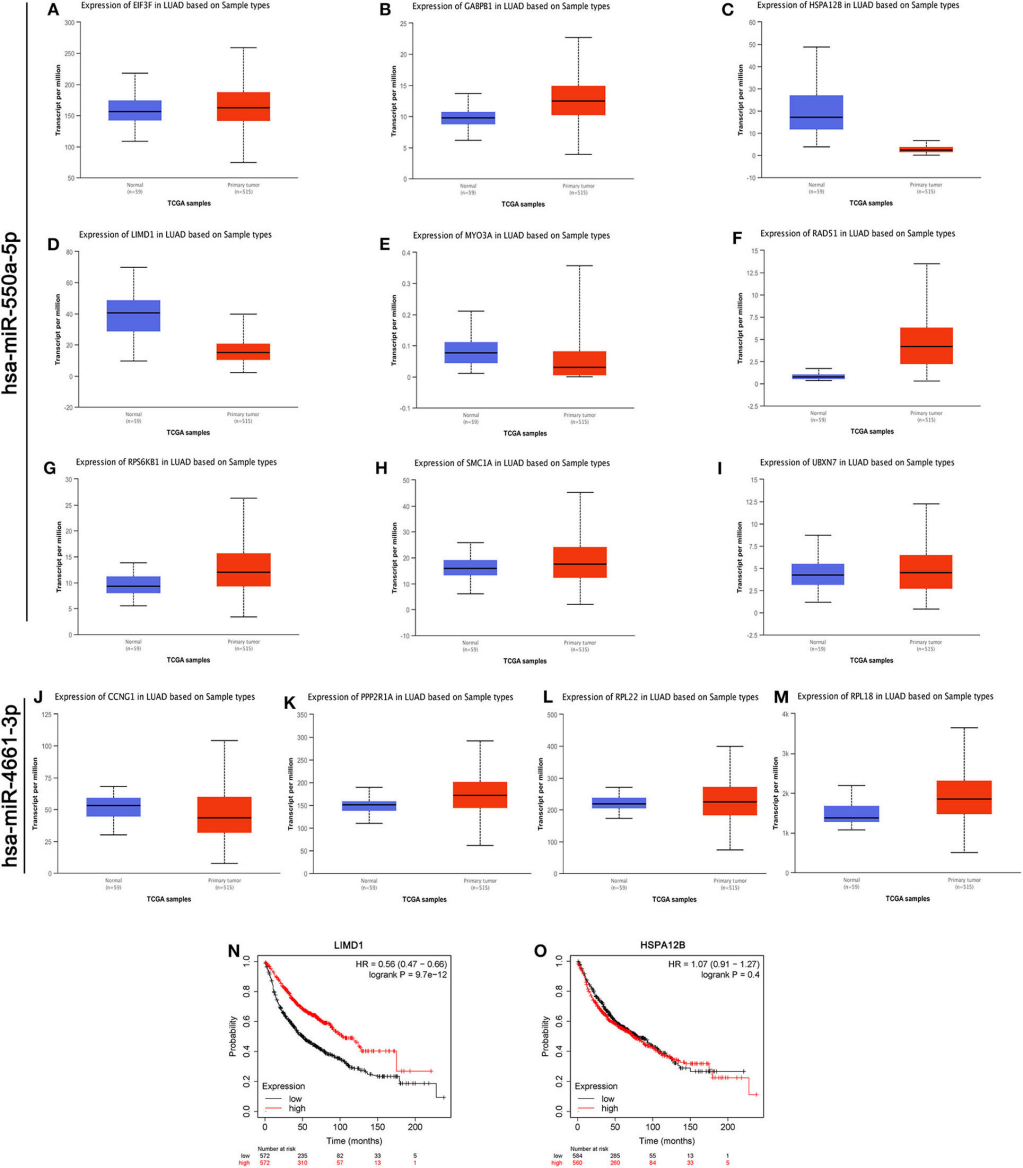
Evaluations of the hub genes of miR-550a-5p. **(A–M)** The mRNA expression of hub genes analyzed via UALCAN database. **(N,O)** Kaplan–Meier survival curve of LIMD1 and HSPA12B in LUAD.

As we know, there is a negative regulation between most miRNA and their downstream target genes. Evaluation of the expression of hub genes of miR-550a-5p and miR-4661-3p was conducted by UALCAN, an analysis website based on the TCGA database. However, in UALCAN, no negative correlation between these hub genes and miR-4661-3p was found in LUAD samples. Then, the survival prognosis analysis of genes that were negatively related to miR-550a-5p was performed by Kaplan–Meier Plotter (Figure 4). Since LIMD1 is a tumor suppressor gene in LUAD, we speculated that miR-550a-5p might exert a cancer-promoting effect by directly targeting LIMD1.

### miR-550a-5p Was Up-Regulated Both in LUAD Tissues and in Cell Lines

**Figure 5:**
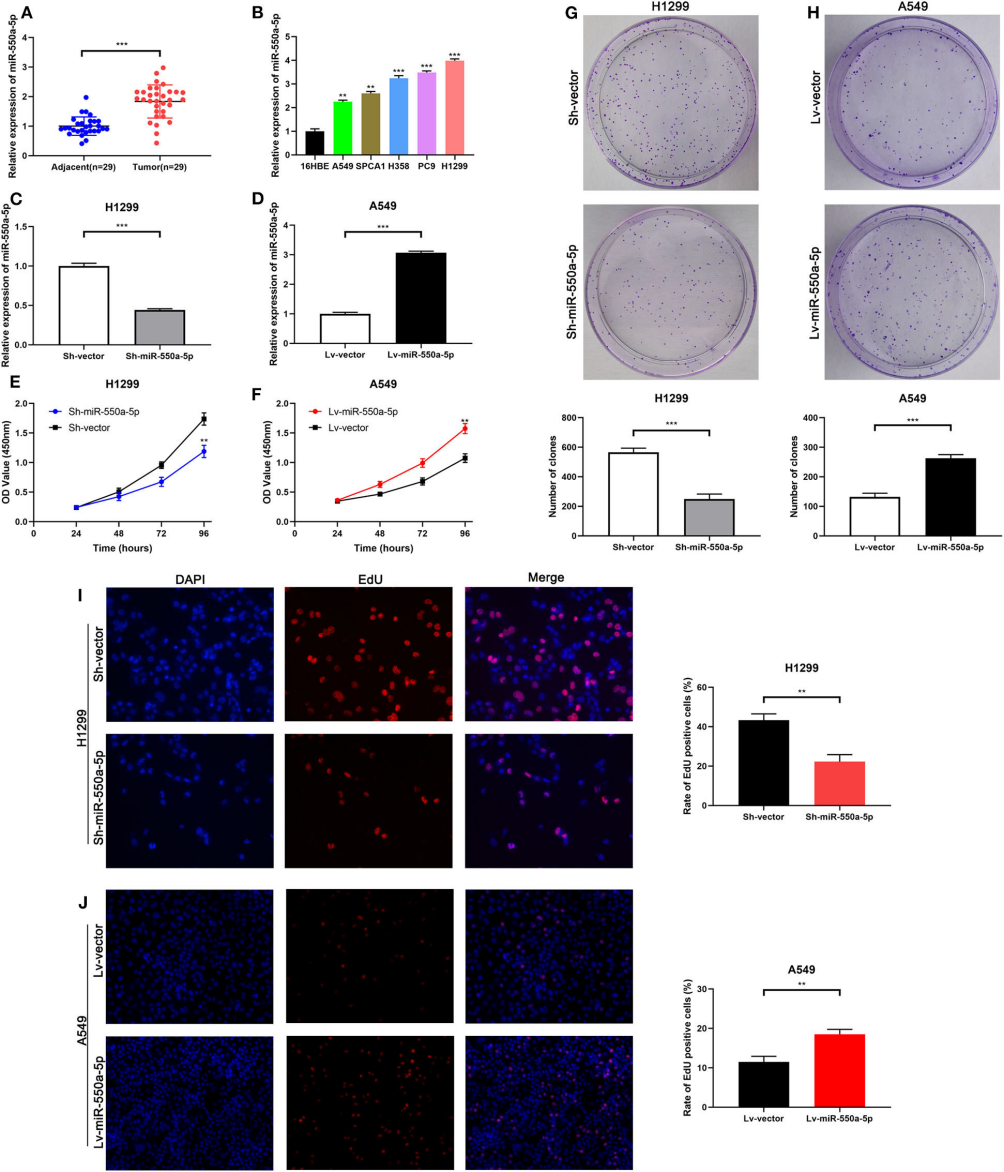
miR-550a-5p accelerated LUAD cell proliferation. **(A)** miR-550a-5p level in 29 LUAD tissues and paired adjacent tissues investigated by RT-qPCR. **(B)** miR-550a-5p level in normal lung epithelial cell line 16HBE and five LUAD cell lines investigated by RT-qPCR; U6 was used as an internal control. **(C,D)** Verification of miR-550a-5p expression in H1299 and A549 cell lines after lentivirus treatment by RT-qPCR. **(E,F)** CCK-8 was used to determine the proliferation of transfected H1299 and A549 cell lines. **(G,H)** Effect of miR-550a-5p on colony-forming capacity of H1299 and A549 cell lines treated with lentivirus. **(I,J)** Representative profile of EdU cell growth in H1299 and A549 cell lines after transfection with lentivirus, respectively, compared with the control. Data expressed as mean ± SD (*P < 0.05; **P < 0.01; ***P < 0.001).

Based on the results of the previous bioinformatics analysis, RT-qPCR detection of the expression of miR-550a-5p in LUAD tissues and cell lines was performed. Compared with the normal bronchus cell line 16HBE, miR-550a-5p was highly expressed in LUAD cell lines, and it was also highly expressed in LUAD tissues compared to adjacent tissues (Figure 5; Table 3). These results were consistent with the bioinformatics analysis.

**Table 3:** Clinical pathological characteristics.

**Characteristics**	**Total**	**miR-550a-5p expression**		***P*-value**
		**High**	**Low**	
Gender				1.0
Male	13	12	1	
Female	16	15	1	
Age (years)			0.483	
≥60	14	14	0	
<60	15	13	2	
Smoker				0.163
Yes	12	10	2	
No	17	17	0	
Tumor size				0.037^*^
T1 + T2	6	4	2	
T3 + T4	23	23	0	

The tumor size was classified by The Union for International Cancer Control (UICC) version 8.

* *P < 0.05*.

### miR-550a-5p Modulated LUAD Proliferation *in vitro*

**Figure 6:**
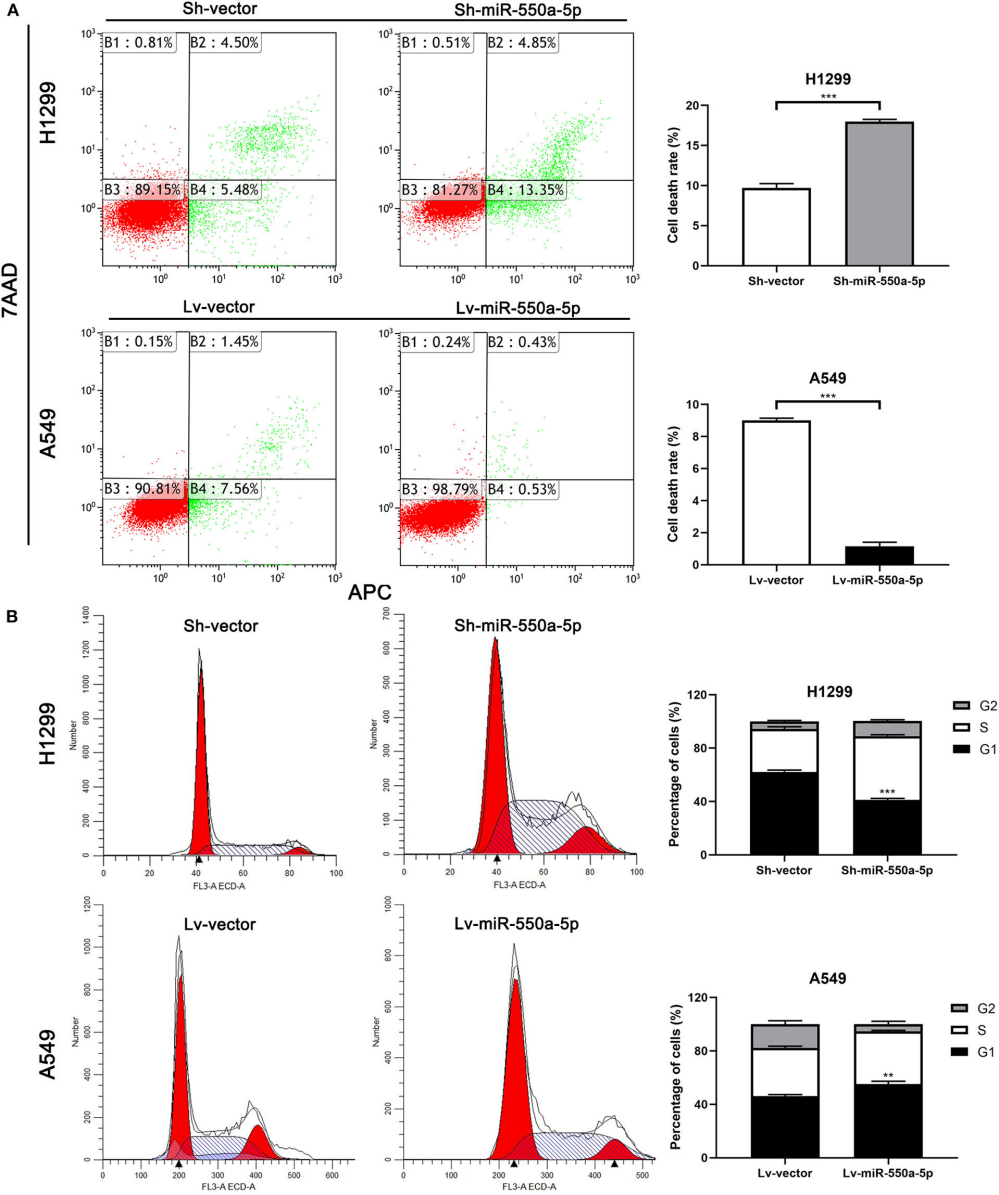
miR-550a-5p affected cell cycle and apoptosis in LUAD cells. **(A,B)** FACS analysis of effect of miR-550a-5p expression alteration on cell cycle and apoptosis. Data expressed as mean ± SD (*P < 0.05; **P < 0.01; ***P < 0.001).

To determine the role of miR-550a-5p in LUAD, loss- and gain-of-function experiments were performed via transfecting Lv-miR-550a-5p into the A549 cell line compared with Lv-vector, as well as sh-miR-550a-5p into the H1299 cell line relative to Sh-vector. The transfection efficiency was verified by RT-qPCR. CCK-8 assay, EdU assay, and colony formation assay showed that up-regulation of miR-550a-5p obviously enhanced A549 cell line proliferation compared with the negative control, while down-regulation of miR-550a-5p in the H1299 cell line showed attenuation (Figure 5). In addition, by regulating miR-550a-5p, the cell cycle and apoptosis of LUAD cell lines were also significantly affected (Figure 6). These experimental results demonstrated that miR-550a-5p exerted a promoting effect on cell proliferation.

### miR-550a-5p Negatively Regulated LIMD1 by Directly Binding to its 3′-UTR

**Figure 7:**
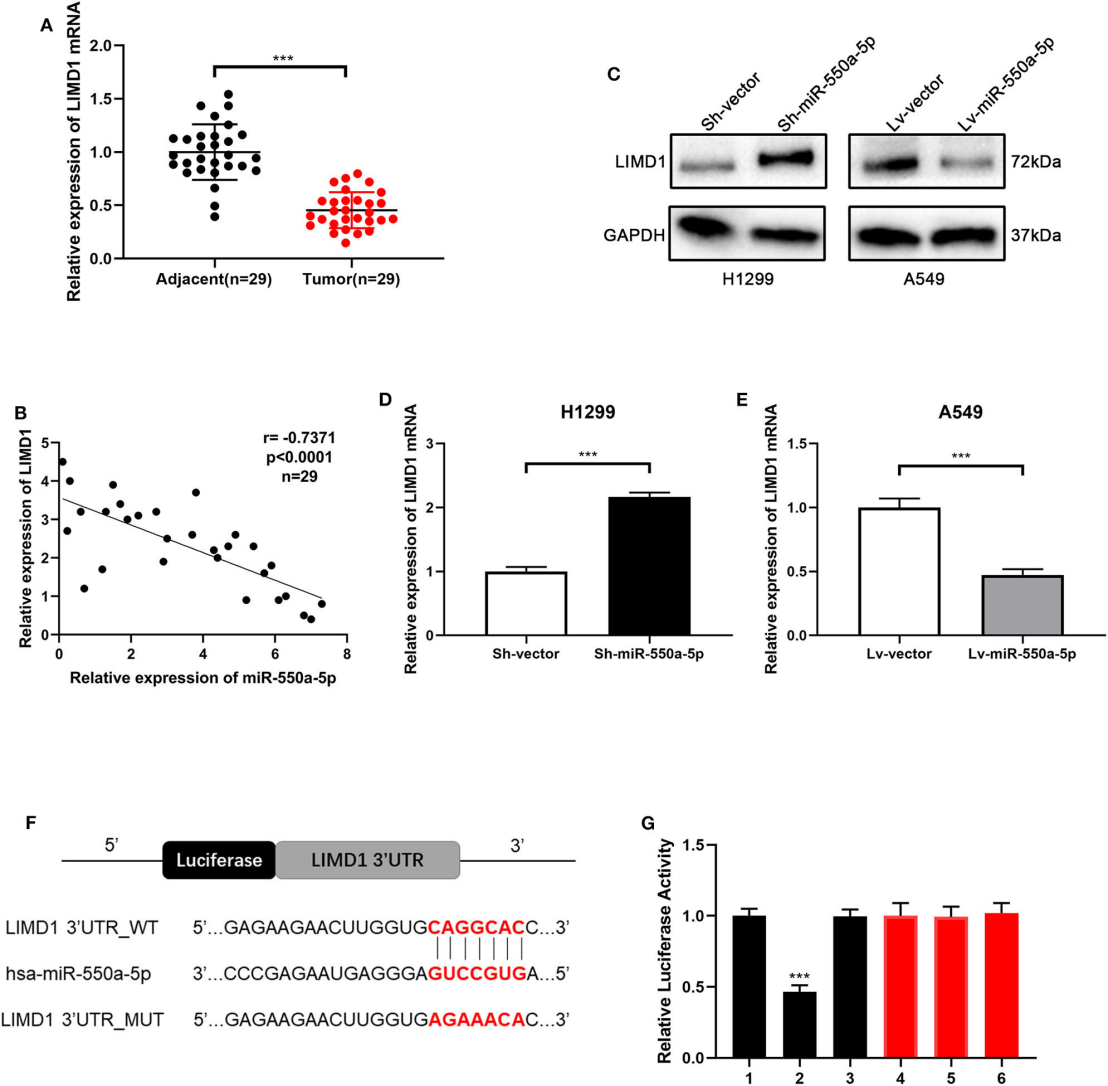
miR-550a-5p negatively regulated LIMD1 by directly binding to its 3′-UTR. **(A)** LIMD1 mRNA in 29 LUAD tissues and paired adjacent tissues was investigated by RT-qPCR. **(B)** A negative correlation was found between LIMD1 and miR-550a-5p in 29 clinical tumor samples. **(C–E)** Regulatory effect of miR-550a-5p on LIMD1 was detected by Western blot and RT-qPCR; GAPDH was used as an internal control for Western blot, while U6 was used for RT-qPCR. **(F)** The potential miR-550a-5p seed region at the 3′-UTR of LIMD1 mRNA. **(G)** A luciferase reporter assay was conducted to verify that miR-550a-5p directly bound to the 3′-UTR sequences of LIMD1. 1: pGL3-LIMD1; 2: pGL3-LIMD1 + miR-550a-5p mimics; 3: pGL3-LIMD1 + NC; 4: pGL3-LIMD1 mut; 5: pGL3-LIMD1 mut + miR-550a-5p mimics; 6: pGL3-LIMD1 mut + NC. Luciferase activity was normalized by the ratio of firefly and Renilla luciferase signals. Data expressed as mean ± SD (*P < 0.05; **P < 0.01; ***P < 0.001).

In order to verify whether LIM domains containing 1 (LIMD1), a hub gene screened out from the bioinformatics analysis above, is the target gene of miR-550a-5p, RT-qPCR of LIMD1 in patients' samples was conducted in the first step. Compared to adjacent tissues, relative expression of LIMD1 mRNA was low expressed in tumor tissues. In addition, Western blot and RT-qPCR experiments were carried out on cell lines treated with lentivirus, and it was found that the expression level of LIMD1 had a negative correlation with miR-550a-5p. For further investigation, a luciferase experiment was performed to verify whether 3′-UTR in mRNA of LIMD1 was a target of miR-550a-5p. The relative luciferase intensity of cells with miR-550a-5p and LIMD1 3′-UTR plasmids was obviously reduced compared with cells transfected with mutation sites of LIMD1 3′-UTR (Figure 7). These results revealed that miR-550a-5p might negatively regulate LIMD1 in LUAD by directly binding to its 3′-UTR.

### Knockdown of LIMD1 Partially Abolished the miR-550a-5p-Mediated Effects

**Figure 8:**
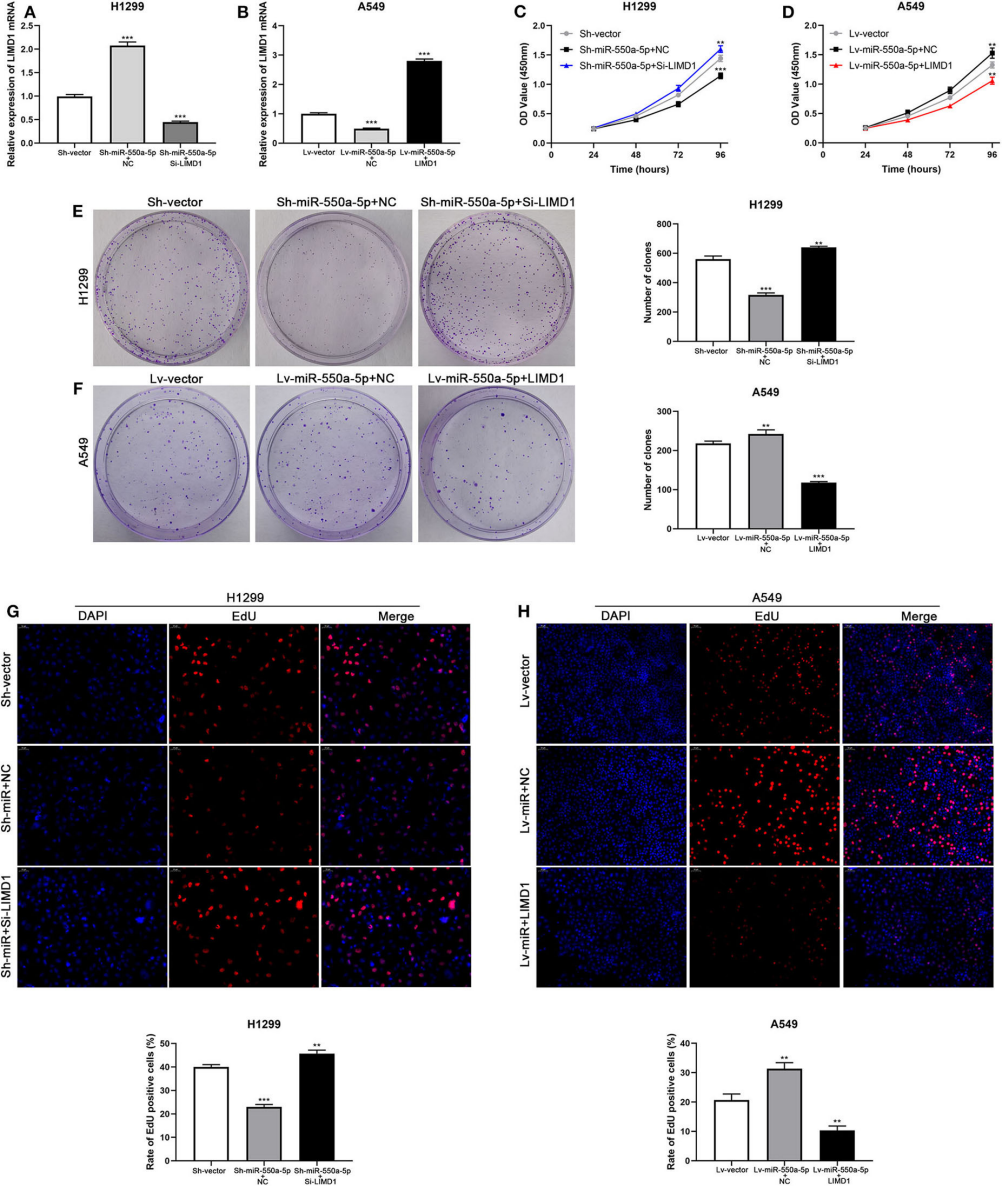
miR-550a-5p accelerated LUAD cell proliferation by targeting LIMD1. **(A,B)** The expression level of LIMD1 mRNA was verified by RT-qPCR in A549 and H1299 cell lines co-transfected by miR-550a-5p and LIMD1. **(C–H)** Rescue assays consisted of CCK8 assay, colony formation assay, and EdU assay were performed to verify the roles of miR-550a-5p and LIMD1 in regulation of LUAD cell proliferation. Data expressed as mean ± SD (*P < 0.05; **P < 0.01; ***P < 0.001).

Cell lines were co-transfected with Lv-miR-550a-5p and LIMD1 or Sh-miR-550a-5p and Si-LIMD1 to perform rescue assays; Lv-vector and Sh-vector were designed as negative controls. The efficiency of transfection was confirmed by RT-qPCR (Figure 8). The results of CCK-8 assay, colony formation assay, and EdU assay suggested that the reverse regulation of LIMD1 could partially abolish the affection of miR-550a-5p on tumor proliferation in LUAD.

### miR-550a-5p Promoted Xenograft Tumor Formation *in vivo*

**Figure 9:**
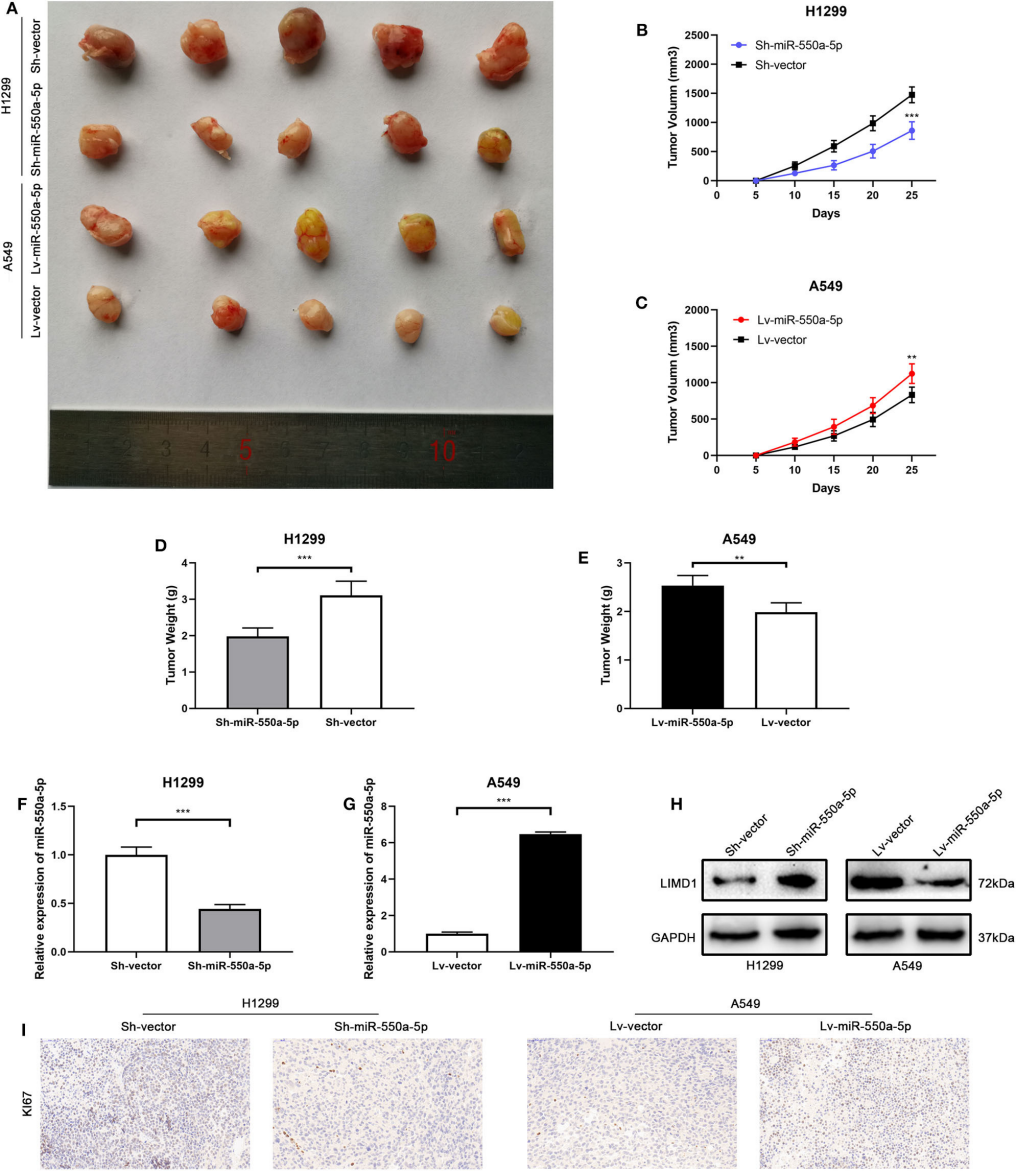
miR-550a-5p promoted xenograft tumor formation. **(A)** Photographs of tumors were obtained from the different groups of nude mice transfected with Lv-miR-550a-5p, Lv-vector and Sh-miR-550a-5p, Sh-vector respectively. **(B,C)** Growth curve of tumor volumes were calculated every 5 days. **(D,E)** Weight of tumors was measured and compared between groups. **(F,G)** The expression level of miR-550a-5p in xenografts was detected by RT-qPCR. **(H)** The relative expression of LIMD1 in xenografts was detected by Western blot. **(I)** The expression level of Ki67 in samples collected from nude mice was analyzed by IHC. Data expressed as mean ± SD (*P < 0.05; **P < 0.01; ***P < 0.001).

To confirm whether miR-550a-5p could also exert a promoting effect on tumor formation *in vivo*, cell lines transfected with lentivirus or empty vector were subcutaneously injected into both sides of the flanks of BALB/C nude mice. The results showed that down-regulated miR-550a-5p decreased tumorigenic ability *in vivo* compared to NC, while up-regulation exhibited an opposite result. In addition, IHC staining against Ki67 was consistent with the result that miR-550a-5p could promote LUAD proliferation (Figure 9). Therefore, these findings demonstrated that miR-550a-5p promoted tumor formation *in vivo*.

## Discussion

Although there was a continuing decline in recent years, it is still one of the leading causes of cancer deaths. The molecular mechanism in the development of lung cancer is still lacking (6). LUAD is the most common type of lung cancer and is one of the most aggressive and rapidly fatal tumor types, accounting for half of all lung cancer cases (7).

As far as the current research stage is concerned, miRNAs play an important role in the tumor, functioning as diagnostic or prognostic biomarkers for malignancies and being potential therapeutic targets (8–10). However, the specific biological function of most abnormally expressed miRNAs in LUAD remains unclear (11, 12). Therefore, the research of miRNAs in LUAD still needs to be supplemented and improved.

In recent years, with the advent of the era of data technology, bioinformatics analysis of various databases has become popular. In this study, based on the TCGA database, some miRNAs were found up-regulated in LUAD cases through bioinformatics analysis. Among them, miRNAs reported in LUAD-associated articles and without difference in 5-year survival prognosis were ignored. Therefore, miR-550a-1, miR-550a-2, and miR-4661 were screened out for further study.

It comes to light that most miRNAs are transcribed as a long precursor by RNA polymerase II and undergo extensive processing before they are integrated into the active RISC (13). Initially, primary miRNAs (pri-miRNAs) turn into precursor miRNAs (pre-miRNAs) by getting trimmed by Drosha to a hairpin duplex in the nucleus. Subsequently, the duplex gets transported to the cytoplasm and integrated into Ago2 protein. During the process of strand selection, one passenger strand of the strands of the duplex is discarded, leaving a guide strand with Ago2 to form activated RISC (14, 15). The RISC allows the guide strand to interact with the target mRNA, usually with its 3′-UTR. This interaction results in translational repression by the degradation of the target mRNA (16–20).

Speaking of the guide strand and passenger strand, for mankind, guide strand has an excess of purines (A/G), whereas the passenger strand is an excess of pyrimidines (U/C) (21). Therefore, through the search of miRbase and relative articles, we conducted further research on miR-550a-5p, which is processed from miR-550a-1 and miR-550a-2, and miR-4661-3p formed from miR-4661. According to the node degree of target genes predicted, their hub genes were screened out, and the miR-hub genes network was constructed. Through this network, we found that most of the hub genes could be potentially modulated by miR-550a-5p, and in recent research reports, miR-550a-5p is also involved in the development of colorectal cancer. More importantly, based on UALCAN, the hub genes did not have a negative correlation with miR-4661-3p. Therefore, we chose miR-550a-5p for further research. RT-qPCR assays on cells and tissues were performed, as well as cell function experiments and nude mice tumorigenesis experiments, which proved that miR-550a-5p played an important role in LUAD.

**Figure 10:**
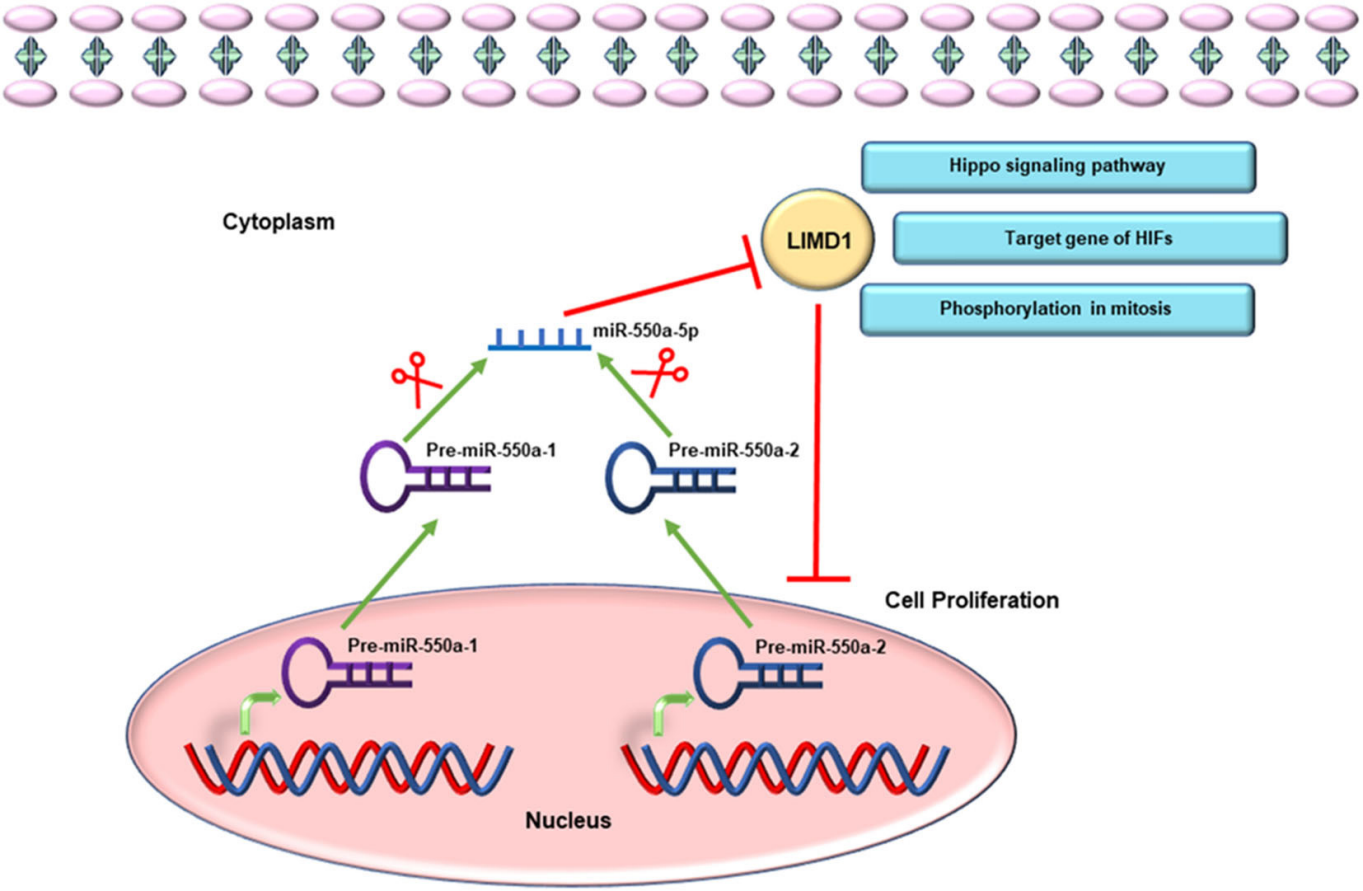
Schematic diagram of miR-550a-5p targeting LIMD1 to modulate cell proliferation in LUAD.

Given the miR-hub genes network and UALCAN database, the expression of LIMD1 and miR-550a-5p exhibited a negative correlation in LUAD. Meanwhile, the survival prognosis analysis of LIMD1 in lung cancer from the Kaplan–Meier Plotter database also showed its significance in tumor inhibition. LIMD1 is a member of the Zyxin proteins, encoded at chromosome 3p21.3 and widely expressed in human tissues (22, 23). A large number of previous studies have suggested that LIMD1 functions as a tumor suppressor (24–27). In terms of molecular mechanism, LIMD1 could participate in cellular processes and pathways through its scaffold function, meaning that the tumor-suppressive function of LIMD1 is likely to be regulated by different signal cascades (28). For example, LIMD1 can regulate Hippo-YAP signaling activity (29), and its phosphorylation is necessary for mitotic progression (23). LIMD1, identified as a hypoxia-inducible factor (HIF) target gene, participates in HIF-associated regulation of tumorigenesis (28). Therefore, through experiments in the current research, directly targeting the 3′-UTR of LIMD1 is the reason that miR-550a-5p functions as a tumor promoter (Figure 10).

In conclusion, a new potential prognostic and therapeutic biomarker, LUAD proliferation-associated miR-550a-5p, has been identified by bioinformatics analysis and experimental validation *in vitro* and *in vivo*, which promotes LUAD by silencing a known suppressor oncogene LIMD1.

## Data Availability Statement

The raw data supporting the conclusions of this article will be made available by the authors, without undue reservation.

## Ethics Statement

This study was approved by the ethics committee of The First Affiliated Hospital of Nanjing Medical University. Patients provided written informed consent to participate.

## Author Contributions

Z-ZG is responsible for experimental design. Y-ZH and Z-JM are responsible for instrument operation. WJ, YX, C-FP, KW, and Y-JS are responsible for data analysis. Y-JC and LC are for providing overall ideas. All authors contributed to the article and approved the submitted version.

## Conflict of Interest

The authors declare that the research was conducted in the absence of any commercial or financial relationships that could be construed as a potential conflict of interest.
